# Neuroprotection in Traumatic Brain Injury: Mesenchymal Stromal Cells can Potentially Overcome Some Limitations of Previous Clinical Trials

**DOI:** 10.3389/fneur.2018.00885

**Published:** 2018-10-24

**Authors:** Marco Carbonara, Francesca Fossi, Tommaso Zoerle, Fabrizio Ortolano, Federico Moro, Francesca Pischiutta, Elisa R. Zanier, Nino Stocchetti

**Affiliations:** ^1^Neuroscience Intensive Care Unit, Department of Anaesthesia and Critical Care, Fondazione IRCCS Cà Granda Ospedale Maggiore Policlinico, Milan, Italy; ^2^Laboratory of Acute Brain Injury and Therapeutic Strategies, Department of Neuroscience, Istituto di Ricerche Farmacologiche Mario Negri IRCCS, Milan, Italy; ^3^School of Medicine and Surgery, University of Milan-Bicocca, Milan, Italy; ^4^Department of Pathophysiology and Transplants, Milan University, Milan, Italy

**Keywords:** traumatic brain injury, mesenchymal stromal cells, brain protection, brain repair, vulnerability

## Abstract

Traumatic brain injury (TBI) is a leading cause of death and disability worldwide. In the last 30 years several neuroprotective agents, attenuating the downstream molecular and cellular damaging events triggered by TBI, have been extensively studied. Even though many drugs have shown promising results in the pre-clinical stage, all have failed in large clinical trials. Mesenchymal stromal cells (MSCs) may offer a promising new therapeutic intervention, with preclinical data showing protection of the injured brain. We selected three of the critical aspects identified as possible causes of clinical failure: the window of opportunity for drug administration, the double-edged contribution of mechanisms to damage and recovery, and the oft-neglected role of reparative mechanisms. For each aspect, we briefly summarized the limitations of previous trials and the potential advantages of a newer approach using MSCs.

## Introduction

Traumatic brain injury (TBI) is the leading cause of mortality and morbidity across all ages in all countries. In Europe, it is estimated that 2.5 million people suffer a TBI each year, 1 million are admitted to hospital, and 57,000 die (1). TBI survivors have to deal with chronic post-injury motor, cognitive, and neuropsychological symptoms/dysfunctions. Even in the milder cases, TBI substantially increases the risk of epilepsy, stroke, and late-life neurodegenerative diseases (1). TBI thus implies a huge burden for patients, their families, and society.

Trauma causes primary damage to the brain by multiple mechanisms, including tearing, shearing, and stretching forces. Consequently, a cascade of metabolic, biochemical, and inflammatory changes is initiated, leading to secondary damage. Then second insults, both intracranial and systemic such as hypoxia, hypotension and intracranial hypertension, may worsen the progression of the injury.

Treatment of TBI patients has not changed much in the last 20 years, consisting only in supportive therapy directed at prevention, early detection and treatment of second insults, since all pharmacological trials testing neuroprotective agents have failed (2–5). This translational defeat may have several explanations, analyzed in numerous papers (6–8). In these critical reappraisals, many factors were identified at preclinical and clinical levels as area of improvement. They included, but were not limited to, pharmacokinetics and pharmacodynamics, inadequate sample sizes, heterogeneity of TBI populations, the lack of relevant mechanistic early endpoints and insensitivity of global outcome measures (9).

Mesenchymal stromal cells (MSCs) may offer a promising strategy, with preclinical data showing that MSCs of human origin protect the injured brain by acting on multiple mechanisms of protection and repair (10–13), with potential advantages in terms of therapeutic window.

After initial expectations about the possibility of MSC trans-differentiation through neuronal lineage for brain reconstruction, decades of experimental data mainly show that MSCs do not protect the TBI brain through cell replacement, but by stimulating neuroprotective and endogenous neuroreparative mechanisms that this narrative review will discuss. We shall focus on three flaws of past trials that MSC-based therapy has the potential to overcome: the “window of opportunity” for drug administration, the double-edged contribution of mechanisms to damage and recovery, and the important, but often neglected, role of reparative mechanisms.

## Window of opportunity for pharmacological neuroprotection in TBI

The biochemical mechanisms of progressive brain damage are set in motion immediately after TBI as a consequence of the external force applied to the head. Using microdialysis in a rodent model of concussion, Katayama demonstrated a surge of extracellular potassium in the first minutes after injury, parallel with massive release of glutamate—up to 10–100 times the normal concentration (14). The time-resolution of the method, however, was limited (1 min of dead space for dialysate collection); when electrodes were used, almost immediate K+ release was demonstrated after trauma (15).

Early mechanisms of cellular injury act in minutes-to-hours after injury. The massive release of excitatory neurotransmitters, spreads energy failure and overload of free radicals from the contused tissue to surrounding brain regions. Energy crisis alters cell permeability, causing calcium inflow, which triggers mitochondrial dysfunction, with consequent energy failure, and apoptotic/necrotic death. Primary axotomy is uncommon, even in the case of traumatic axonal injury; the alteration of membrane permeability induces edema and impairs axonal transport, making axons more vulnerable to secondary axotomy and demyelination. These cascades clearly indicate how mechanical forces applied to the brain may evolve and propagate to healthy, potentially salvable tissue (16, 17).

The progress of secondary injury, in its sequence of deleterious events over time, is the theoretical basis for neuroprotective strategies. When neuroprotectant drugs were tested under experimental conditions, it became evident that their maximum potential was exploited by early administration or—when possible—by pre-treating the brain before insults (18). In general, however, later exposure to a protective compound gave less or no benefit (19–21).

These findings shaped the design of clinical trials, where drugs had to be administered in the first hours after injury, when there was felt to be a “window of opportunity.” A recent review (5) of 16 robust trials testing neuroprotective agents in TBI indicated a window of opportunity of 4 h in three, 6 h in two, 8 h in seven, and 12 h in one trial, while only three trials tested treatment up to 24 h after injury (Table 1). This narrow window of opportunity makes clinical trials more challenging, reducing enrolment rates and increasing complexity. Patients need to be rescued, stabilized, centralized to the study center, evaluated clinically and by imaging; relatives must be contacted for consent procedures so, finally, randomization and drug preparation and administration can start within the few hours permitted by the protocol. It is no surprise that a number of cases failed to meet the time limit: in several trials ~20% of potential candidates could not be enrolled because of the narrow time window (36). In the same trials the duration of the pharmacological intervention was limited to the first days after ICU admission (Table 1).

**Table 1:** Major randomized clinical trials (RCT) evaluating pharmacological treatments for acute moderate/severe TBI.

**Study**	**Drug**	**Treatment start**	**Treatment duration**	**Sample size**	**Results**
Skolnick (22)	Progesterone	< 8 h	5 days	1,195	ND
Wright 4	Progesterone	< 4 h	4 days	882	ND
Shakur (23)	Anatibant	< 8 h	4 days	228	ND
Marmarou (24)	Bradycor	< 12 h	5 days	139	ND
Eurogroup (25)	Nimodipine	< 24 h	7 days	852	ND
Teasdale (26)	Nimodipine	< 24 h	7 days	352	ND
Perel (27)	Tranexamic acid	< 8 h	1 day	170	ND
Robertson (28)	Erythropoietin	< 6 h	14 days	200	ND
Maas (29)	Dexanabinol	< 6 h	1 day	861	ND
Yurkewicz (30)	Traxopodil	< 8 h	3 days	404	ND
Morris (31)	Selfotel	< 8 h	4 days	693	ND
Marshall (32)	Tirilazad	< 4 h	5 days	1,120	ND
Young (33)	Pegorgotein	< 8 h	1 day	463	ND
Asehnoune (34)	Steroids	< 24 h	10 days	336	ND
Edwards 2	Steroids	< 8 h	2 days	10,008	ND
Grumme (35)	Steroids	< 4 h	8 days	396	ND

*Study selection was derived from the systematic review by Bragge et al. 5. An RCT was defined as robust if it was multicenter, included more than 100 patients, and had low risk of bias. For each study, it is reported first author and year of publication, the investigated drug, treatment start and length, number of patients included, and the effect on outcome, as mortality for Shakur, Perel and Asehnoune and Glasgow Outcome Scale for the others. ND, no statistical difference between intervention and control regarding selected outcome*.

However, mounting evidences indicate that pathophysiologic processes caused by the initial injury do not exhaust themselves in the first days but persist for months or years, and that TBI survivors are at risk of late neurodegenerative diseases (including Alzheimer's and Parkinson's disease, chronic traumatic encephalopathy) (37). For example, Johnson et al. reported immunohistochemical evidence of microglia activation and white matter degradation in subjects who died many years after TBI (38); and in patients there is a relation between chronic inflammation detected by positron emission tomography, up to 17 years post-TBI, and worse cognitive outcomes (39).

Inflammation is an important beneficial mechanism for clearing pathological debris and effecting repair (40–44); however, if dysregulated, it may also contribute to neuronal damage. The relative positive or negative effects of inflammation in relation to time from injury are still far from certain, and a threat of neurodegeneration associated with late microglia inhibition has recently been reported in TBI subjects chronically treated with minocycline, an antibiotic that can inhibit microglia activation (45). With this in mind, immunomodulatory rather than inhibitory strategies contributing to the resolution of inflammatory changes may prove effective, with a wide therapeutic window.

MSCs have high immunomodulatory potential both *in vitro* and *in vivo*. It has been suggested that in response to injury these cells can sense the injured environment, leading to the promotion of injury resolution and regenerative processes through the secretion of immunomodulatory bioactive factors and trophic molecules including growth factors, cytokines, and antioxidants (46–48), that may vary in relation to the needs of the tissue and the time from injury.

Preclinical studies in rodents address the effects of MSCs in a wide range of TBI-to-therapy intervals, with consistent data from several laboratories showing their efficacy when infused 24 h post-TBI. Both central (49–60) and systemic (60–75) administration of MSCs 24 h post-TBI have resulted in early and persistent improvements of functional and structural outcomes. It was recently shown that a double systemic infusion of MSC (at 4 and 24 h) post-TBI was more effective than a single dose at 24 h (76). The authors showed 4 and 24 h post-TBI peaks of IL1β, TNFα, and IFNγ, suggesting that a shorter lag time between TBI and treatment may be important to counteract early pro-inflammatory changes. Whether this gain in protection was due to multiple doses or the earlier treatment still needs to be fully investigated.

MSC infusion has also given protective effects when delivered in the sub-acute phase (between 2 and 7 days after injury) either systemically (77, 78) or centrally (79–81). Kota et al. administered bone-marrow MSCs (BM-MSCs) 3 days after TBI, showing IFN-γ and TNF-α reductions of ~50% (82), with significant inhibition of brain permeability, edema, microglial activation and systemic levels of norepinephrine, while promoting neurogenesis (83).

Effect size relative to the time from experimental TBI to MSC administration has been evaluated in a recent meta-analysis (10). The analysis confirms MSC efficacy when infused from 2 h up to 7 days, with no significant differences in effect sizes relative to the time from TBI to intervention.

So far there are only few reports of MSC given in the chronic phase of TBI. At 2 months post-TBI, MSC transplant into the lesion core improved sensorimotor deficits and promoted neuro-restorative processes (84, 85). However, at this stage iv injection was not effective (86), suggesting that MSC may act through complementary mechanisms when infused locally into the brain or systemically, the latter no longer being sufficient at later stages.

Compelling data on MSC rationale, efficacy, immune tolerance, and feasibility are fostering the design of clinical studies. Pragmatically, to optimize the reduction of toxic cascades and the promotion of endogenous reparative mechanisms, an administration of MSC within 48 h from TBI would seem to be desirable. However, only data from clinical trials will provide a definitive answer in term of the best timing for intervention.

## Double-edged contribution of mechanisms to damage and recovery

Counteracting specific damage pathways should reduce the extent of tissue injury, and ultimately contribute to a better outcome. This is the logic behind several lines of investigation in TBI, from studies on calcium blockers to N-methyl-D-aspartate (NMDA) antagonists.

Under physiological conditions, glutamate plays an important role as a neurotransmitter; it is also involved in coupling glucose utilization and neuronal activity (87). However, high concentrations of glutamate (100–500 micromoles) are lethal for neurons *in vitro*, and have been measured in the extracellular space of experimental TBI in rodents. In humans too there is evidence of high extracellular concentrations of glutamate after TBI, particularly in the elderly (88). This supported the hypothesis that blocking glutamate receptors (like the NMDA subtype) might attenuate the deleterious effects of the glutamate surge induced by TBI. Several compounds inhibiting the NMDA receptors, with competitive or non-competitive mechanisms, have therefore been tested in experimental and clinical settings. While in the laboratory evidence of neuroprotection was found (89, 90), clinical trials all failed to show benefit (91). Among the possible explanations for these repeated failures, there is the hypothesis that NMDA receptor activity is essential for neuronal function and integrity, so that NMDA blockage at critical time points (92), especially in vulnerable phases after TBI, could be deleterious rather than protective.

Another failed neuroprotective treatment is corticosteroids, which were tested in the first mega-trial in TBI at the end of the last century, “Corticosteroid randomization after significant head injury” (CRASH) (2). The hypothesis leading to this trial was that inflammation is a key component of the brain response to TBI and that blocking the inflammatory cascade could therefore be protective. Soluble mediators and cellular components of inflammation were investigated and related to the extent of brain damage. Unfortunately, however, the CRASH results showed no improvement in favorable outcomes and there was in fact a higher risk of mortality in the treatment group, not fully explained by systemic complications (such as infection and gastric bleeding); this may call into play the complex double-edged function of inflammation involved not only in toxic but also in regenerative processes, as discussed above. In this context MSCs affect the biology of the injured cells and tissue through the secretion of cytokines, morphogens, small molecules, and cargo-bearing exosomes (93, 94), which skew the activation of immune cells from a toxic to a more permissive phenotype, thus contributing to injury resolution and tissue repair (55, 58).

## The neglected role of reparative mechanisms

Besides toxic cascades TBI also induces neuro-restorative processes including neurogenesis, gliogenesis, angiogenesis, synaptic plasticity, and axonal sprouting (95–97). These events are induced by biochemical factors such as growth factors, steroids, and neurotransmitters, released in response to injury, with the potential for counteracting progression of the injury and contributing to functional recovery. However, all these spontaneous processes are short-lived and the efficacy of the self-repair responses is limited. Providing the injured tissue with a facilitatory milieu that increases endogenous reparative mechanisms may open up new therapeutic opportunities.

In the adult brain the subventricular zone (SVZ) and the subgranular zone (SGZ) of the dentate gyrus (DG) are populated by neural stem cells, which can differentiate into functional neurons (95, 98). Proliferation in the DG is age-dependent, with higher potential in the juvenile brains. The new cells can differentiate into functional mature neurons, involved in higher functions.

The neurogenic response after TBI comprises three phases: proliferation of precursors/progenitors cells, migration to injured tissue, and differentiation into proper cell types (99). An increased proliferative response in the hippocampus 2 days after TBI, with a peak in the first week after injury, has been described in different TBI models (100). These proliferating cells may differentiate into astrocytes, oligodendrocytes, and neurons, and extend projections alongside the hippocampal mossy fibers participating in recovery of function.

TBI induces a proliferative response in the neurogenic niche in the SVZ and hippocampus (101), under stimulation by growth factors. Preclinical studies have shown that intracerebral administration of single growth factors including fibroblast growth factor 2 (FGF-2) and epidermal growth factor (EGF) can promote endogenous neurogenesis after TBI (102, 103) and improve cognitive outcome. Similarly, infusion of VEGF into the lateral brain ventricle in TBI mice promotes cell proliferation in the SVZ and the peri-lesional cortex after TBI (104); VEGF in fact mediates the survival of newly generated neurons rather than proliferation of neuroblasts (105).

On account of their neurogenic and neuroprotective effects, growth factors are an interesting tool to stimulate reparative processes after TBI. However, their administration after injury is linked to temporal issues related to their rapid kinetics and limited effects. Studies in TBI rodents have shown increased amounts of growth factors after MSC treatment (52, 55, 64, 106–108), leading to the promotion of endogenous restorative processes and suggesting that MSCs may act as a local bioreactor able to produce and release a multitude of growth factors, depending on the specific requirements of the injured tissue.

It has been shown that MSCs stimulate endogenous neurogenesis with an higher proliferation rate in the SVZ and SGZ (64) and an increased number of developing neurons in the SVZ (detected as doublecortin marker) (57, 60); they also stimulate axonal regeneration, as documented by increased GAP-43 expression (58, 107) in MSC-treated TBI animals. Likewise, their ability to promote plasticity in TBI has been documented by infusing a fluorescent dye into the contralateral cortex 5 weeks after injury and measuring its transport from the injection site to the injured hemisphere through the corpus callosum 1 week later (79). Functional outcome and axonal fiber length were increased in MSC-treated animals, suggesting an MSC mediated effect on neuronal connectivity by directing axonal projections, neurite outgrowth and elongation in the injured cortex.

Another aspect linked to neuroplastic processes is represented by glial activation and extracellular matrix composition, both aspects possibly being modulated by MSCs. Acute glial activation is needed to clear excessive glutamate release and remove cellular debris (109, 110). However, at chronic stages, excessive gliotic scar may hamper remodeling processes (111). MSCs reduce the gliotic scar surrounding the contusion 1 month after TBI and this effect is associated with a smaller lesion and better functional recovery (55, 57). MSCs can also alter the extracellular matrix composition, allowing restorative plasticity by circuit reorganization (112).

MSCs act also on vascular cerebral compartment, increasing vessel density in the pericontusional tissue after acute (24 h post-TBI) (57, 60, 113, 114), sub-acute (7 days) (115), and chronic (84) administrations. This suggests that rescue effects on injured vessels as well as regenerative action on brain vasculature involve mechanisms stimulated by cell therapy. In fact, gene expression microarray analysis showed MSC expression of genes involved in angiogenic processes possibly sustaining both neurovascular repair in the acute phase after injury and neovascularization later on (81).

## Conclusions

Despite its high prevalence and heavy social burden, TBI remains a neglected syndrome. Acute care for TBI patients relies on maintenance of cerebral and body homeostasis, blunting or avoiding further insults. After more than 30 years looking for treatments, broadly defined as neuroprotective strategies, to reverse or mitigate injury progression in TBI, we still lack any effective therapy. The reasons for this failure have been extensively analyzed, and new therapeutic approaches for dealing with them could have higher translational potential. Experimental studies support the hypothesis that MSCs may overcome three of the major limitations. First, MSCs in animal models show efficacy when administered within the acute, sub-acute and delayed phases post-TBI, not being limited by a narrow window of opportunity. Second, preclinical data support the notion that MSCs influence a complex pathway such as inflammation, favoring restorative over deleterious aspects. Finally, the recognition that MSCs act on the injured environment fostering reparative processes, relies on a new paradigm, exploitation of the endogenous ability of self-repair.

In conclusion, MSCs have the potential to be the next candidate for neuroprotective trials in TBI patients.

## Authors contributions

MC, TZ, ERZ, and NS designed the review, assembled a preliminary draft, and incorporated further contributions from each author into subsequent versions. All the authors revised it critically for important intellectual content and approved the final version.

### Conflict of interest statement

The authors declare that the research was conducted in the absence of any commercial or financial relationships that could be construed as a potential conflict of interest.
